# Cinnamon Shows Antidiabetic Properties that Are Species-Specific: Effects on Enzyme Activity Inhibition and Starch Digestion

**DOI:** 10.1007/s11130-019-00760-8

**Published:** 2019-08-01

**Authors:** Nicholas J. Hayward, Gordon J. McDougall, Sara Farag, J. William Allwood, Ceri Austin, Fiona Campbell, Graham Horgan, Viren Ranawana

**Affiliations:** 1 The University of Aberdeen, Rowett Institute, Foresterhill, Aberdeen, AB25 2ZD Scotland; 2 The James Hutton Institute, Invergowrie, Dundee, DD2 5DA Scotland; 3 Biomathematics & Statistics Scotland, Aberdeen, AB25 2ZD Scotland

**Keywords:** Cinnamon, Species, Anti-diabetic, Enzyme inhibition, Starch digestibility

## Abstract

Despite considerable research the evidence around the antidiabetic properties of cinnamon remains equivocal, and this may be due to varietal differences which is an aspect that is understudied. This study systematically compared the anti-hyperglycaemic properties of the four major commercial cinnamon types used around the world (Chinese; *Cinnamomum cassia* [CC], Indonesian; *C. burmanii* [IC], Vietnamese; *C. loureirii* [VC], and Ceylon; *C. zeylanicum* [SC]). LC-MS analysis showed distinct diffrences in the phytochemical profiles of cinnamon with SC showing the lowest coumarin concentration. CC and IC had the highest polyphenol levels and antioxidant potential, and all four types differed significantly in their content (*P* < 0.001). All cinnamon types showed potent species-specific effects on starch digestion enzyme activity inhibition (*P* < 0.001), CC was most effective against α-amylase and all four strongly inhibited α-glucosidase compared to acarbose. Cinnamon significantly reduced starch breakdown during oral (*P* = 0.006) and gastric (*P* = 0.029) phases of gastro-intestinal digestion with IC and SC showing consistent effects. No effects of cinnamon were seen in the intestinal phase. IC, VC and SC showed the greatest potential to inhibit formation of advanced glycation endproducts (AGEs) during digestion. In conclusion, cinnamon demonstrates anti-hyperglycaemic properties, however effects are species-specific with best overall properties seen for Ceylon cinnamon.

**Electronic supplementary material:**

The online version of this article (10.1007/s11130-019-00760-8) contains supplementary material, which is available to authorized users.

## Introduction

Dietary approaches for managing hyperglycaemia centre around regulating carbohydrate digestion, absorption and glucose uptake rates. Much research has been devoted to natural products that can modulate these mechanisms as they are considered safer and more economical than drugs, and cinnamon has been studied extensively in this regard driven by its use as an antidiabetic agent in traditional medicine systems. *In vitro* studies suggest cinnamon exerts antidiabetic effects through inhibiting gastro-intestinal enzymes, modulating insulin response and sensitivity, improving glucose uptake, inhibiting gluconeogenesis and increasing glycogen synthesis [1]. Human studies assessing *in vivo* effects of cinnamon have shown mixed results [2]. Therefore, there is yet no clear consensus on the antidiabetic potential of cinnamon [3], and this highlights the importance of further research.

The lack of clear evidence could be due to the diverse test materials used in studies, which vary in type (bark powder, water extracts, distillates), quantity and variety. Cinnamon variety in particular has received little research attention, and this is evident from the number of studies assessing antidiabetic effects with no mention of the variety used [4]. The genus cinnamomum consists of over 250 varieties of which four are widely used as a spice, Chinese cinnamon (*C. cassia*; CC), Indonesian cinnamon (*C. burmanii*; IC), Vietnamese cinnamon (*C. loureiroi*; VC) and Ceylon cinnamon (*C. zeylanicum*; SC). To date no studies have attempted to systematically compare the antidiabetic properties of these four cinnamon types, despite evidence of differing compositions. A clearer understanding of their relative antidiabetic properties would be beneficial for better elucidating the potential of cinnamon as an antidiabetic agent. The objective of this study was to compare the anti-hyperglycaemic properties of the above four cinnamon types, focusing on properties related to reducing carbohydrate digestion. The specific aims of the study were to compare the bioactive, polyphenol and antioxidant profiles of cinnamon extracts, their effects on *α*-amylase and *α*- glucosidase enzyme, and their impact on the glycaemic potential of white bread during simulated gastro-intestinal digestion. Advanced glycation endproducts (AGEs) exacerbate diabetic complications through increasing oxidative stress, inflammation and islet cell injury, and there is increasing focus on reducing their incidence. Both endogenously produced and dietary AGES are significant contributors to circulating pools and natural products have been shown to curtail their generation [5]. Natural products can also help reduce AGEs generation in the gastro-intestinal lumen during digestion [6]. Therefore, a secondary aim was to assess the qualitative effects of cinnamon on advanced glycation endproducts (AGEs) generation during gastro-intestinal digestion. The study hypothesised that the biochemical and functional characteristics of cinnamon will be species-specific, and therefore also their anti-hyperglycaemic properties.

## Materials and Methods

### Test Material and Preparation of Extracts

Cinnamon bark powders were purchased from commercial suppliers (CC: Biovea Natural Foods, Scottsdale, AZ, USA; IC: Garrfen Ltd., Bradford, UK; VC: Costco Wholesale Corporation, Crick, UK; SC: Buy Whole Foods Online, Ramsgate, UK) and used to prepare freeze dried aqueous extracts as described by Cheng *et al*. [7]. Briefly, bark powders were dispersed in 75% aqueous ethanol (1:7), heated at 60 °C for 60 min, filtered through muslin and glass sinters, rotary evaporated and freeze dried.

### Characterisation of Extracts Using Liquid Chromatography- Mass Spectrometry (LC-MS)

Freeze dried cinnamon extracts were completely soluble at 1 mg/ml in 5% acetonitrile/ultra pure water (UPW) containing 0.1% formic acid (FA). Samples (20 μL volume equivalent to 20 μg dry weight) were analysed on an LCQ Fleet Ion Trap mass spectrometer (Thermo Scientific Ltd., Hemel Hempstead, UK) attached to an HPLC system consisting of an Accella 600 quaternary pump and Acella photodiode array PDA detector (PDAD) and autosampler. The PDAD scanned three channels at 280, 365, and 520 nm. Samples were eluted on a gradient of 5% acetonitrile (0.1% FA) to 40% acetonitrile (0.1% FA) on a Synergi Hydro C18 column (2.0  × 150 mm; Phenomenex Ltd., Macclesfield, UK) over 30 min at a flow rate of 200 μL/min. The mass spectrometer used an electrospray ionization interface and the samples were analysed in negative-ion mode with two scan events: full-scan analysis followed by data-dependent MS/MS of the most intense ions (normalised collision energies of 45% arbitrary units) in wideband activation mode. Components were identified using published MS and MS^2^ data [8]. Peak areas at 280 nm for the major peaks were determined from four replicate injections using resident Xcalibur™ software (Thermo Scientific Ltd) and were shown as averages ± standard deviation. For simplicity, the more minor proanthocyanidin (PAC) peaks were collected as one measurement.

### Analysis of Total Polyphenols and Antioxidant Potential

Total polyphenol content was measured as described earlier [9]. Cinnamon extracts reconstituted in 50% DMSO were used with gallic acid as the standard. The ferric reducing antioxidant potential (FRAP) was measured as previously described [9] with iron (II) sulphate (FeSO_4_) as the standard.

### Analysis of *α*-Amylase Enzyme Activity Inhibition

The method described by McDougall *et al* [10] was used with minor modifications. Potato starch (Sigma, S2004) was used as the substrate and acarbose as a positive inhibition control. The absorbance data was used to calculate enzyme inhibition rates and IC_50_ values as follows;

$$ Rate\ of\ absorbance\ change\left( Ab{s}_{410}/\min \right)=\left(\frac{{\left[P\right]}_{25}-{\left[P\right]}_0}{\delta t}\right) $$

Where, [P]_25_ = absorbance after 25 min, [P]_0_ = absorbance at 0 min and δt = change in time. The percentage inhibition in the presence of cinnamon or know inhibitor was calculated as follows;

$$ Inhibiton\ of\ absorbance\ change\left(\%\right)=100-\left[\left(\frac{Rate}{Control\ rate}\right)\times 100\right] $$

Where, Rate = rate with inhibitor/sample, Control rate = rate in control. Inhibitor concentrations were plotted against calculated % inhibitions, to produce a linear regression trend line, and the equation for the line used to calculate IC_50_ values.

### Analysis of *α*-Glucosidase Enzyme Activity Inhibition

The method of Watanabe *et al*. [11] was used with minor modifications. *p*-Nitrophenyl-*α*-D-glucopyranoside (5 mM) was used as the substrate and acarbose as the positive control. Enzyme inhibition rates and IC_50_ values were calculated from the absorbance values as described above for the *α*-amylase inhibition assay.

### Effect of Cinnamon Extracts on *In Vitro* Glycaemic Potential of White Bread

The impact of cinnamon extracts on the glycaemic potential of white bread was measured using a validated static *in vitro* model that mimics human gastro-intestinal digestion [12], adapted for measuring glycaemic potential [13]. White bread was prepared using a standard recipe (500 g strong white wheat flour, 22 g sunflower oil, 11 g sugar, 9 g yeast, 9 g salt, 333 g water) in a bread maker (SD-2500, Panasonic, Bracknell, UK). Available carbohydrate content in the bread was measured using a commercial assay (K-ACHDF, Megazyme, Wicklow, Ireland).

The *in vitro* model consisted of oral, gastric and intestinal phases of digestion. The compositions of the digestive solutions used are reported in supplementary material Table 1. Incubations were carried out in 125 ml screw-top specimen pots (order number 216–1824, VWR International, Leicestershire, UK) in a shaking incubator at 150 rpm (Stuart Orbital Incubator, SI50, Cole-Parmer, Staffordshire, UK). Bread samples containing 0.8 g of available carbohydrates were weighed into the specimen pots and 4 mL of cinnamon extract solution (3 mg of extract/mL) added. Simulated salivary fluid (20 mL) was added into all the pots, and 500 μL aliquots of digesta removed into tubes containing 2 mL ethanol and 50 μL 0.1 M HCl for measuring reducing sugars and advanced glycation endproducts (AGEs) respectively. The oral phase was initiated by placing the digestion pots in the shaking incubator at 37 °C for 3 min and terminated with the addition of 19 mL of gastric fluid. At the end of the gastric phase (30 min) the intestinal phase was initiated for 180 min with the addition of 36 mL of intestinal fluid and 123 μL of amyloglucosidase (≥260 U/mL, *A. niger*, Megazyme, Wicklow, Ireland). Further digesta aliquots were removed into ethanol and HCl at the end of the oral and gastric phases, and at 20 min and hourly for 3 h during the intestinal phase. Three independent incubations were carried out for all samples and the data pooled for analysis. Reducing sugars were measured using the dinitrosalicylic acid (DNS) method as previously described following a secondary amyloglucosidase digestion [13].

### Effect of Cinnamon on the Generation of AGEs during Digestion

Fluorescent advanced glycation end-products in the digesta samples were measured as described elsewhere [14]. Samples were spun at 1500 g for 5 min, and the fluoresecence of the supernatant read at 370 nm excitation and 440 nm emission (SpectraMax, GemiXS, Molecular Devices, CA, USA) in 96-well black plates.

### Statistics and Data Calculations

Amounts of rapidly digestable starch (RDS), slowly digestable starch (SDS) and resistant starch (RS) fractions during digestion were calculated as previously described [15]. Statistical analysis was using the Statistical package for the Social Sciences (SPSS; Version 22, IBM Portsmouth, UK). Data on antioxidant potential, polyphenol content, enzyme activity inhibition, reducing sugar contents, RDS, SDS and RS contents were analysed using one-way analysis of variance (ANOVA). *Post-hoc* comparisons where significant differences were observed were carried out using the Tukey procedure. A *p* < 0.05 was considered significant. Data normality was assessed using the Kolmogorov-Smirnov test.

## Results and Discussion

### Characterisation of Cinnamon Extracts by LC-MS

This study comparatively evaluated the antihyperglycaemic properties of aqueous ethanolic cinnamon extracts. Studies have shown that hydro-ethanolic extracts demonstrate the best antioxidant and functional properties compared to fractions obtained by other methods [16]. Although cinnamon bark powder is consumed widely in foods, the small quantities involved are often inadequate to provide physiologically relavent levels of bioactives, which is further hampered by poor bioavailability due to the cellulose matrix. The current study used an ethanol-water fraction to maximise the extraction of organic and water-soluble bioactives and minimise polysaccharide extraction. All four cinnamon types produced dried extract powders with good reconstitution properties. Extract yields varied between types with CC, IC, VC and SC showing yields of 17, 5, 3 and 2% respectively (g dry extract powder/100 g of dry bark powder). The higher extract yield of CC was noteworthy compared to the other three types, and similar levels have been previously observed [17]. Except for SC all other cinnamon types produced a tar-like substance when ethanol was evaporated which may be indicative of high levels of lipophilic compounds. However, all of the extracts freeze dried to a dry free-flowing powder and were readily redissolvable.

**Fig. 1:**
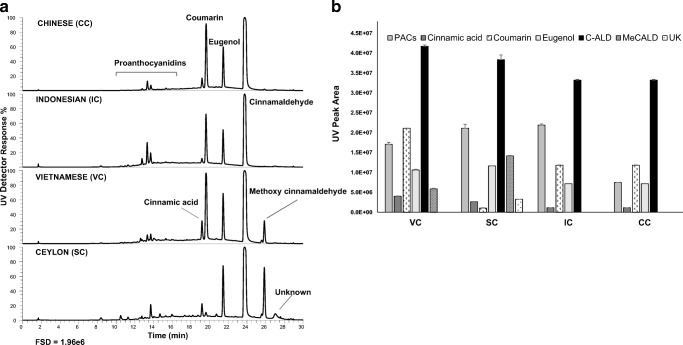
Relative compositions of cinnamon extracts. UV traces of cinnamon extracts (**a**) and composition of the major components (**b**). FSD = full scale deflection of PDAD detector at 280 nm. C-ALD – cinnamaldehyde, MeCALD = methoxy cinnamaldehyde; UK = unknown

The cinnamon extracts showed characteristic compositions by LC-MS (Fig. 1a). The major components in the extracts were cinnamic acid, eugenol, cinnamldehyde, methoxy cinnamaldehyde and coumarin, with lower and variable amounts of proanthocyanidins. From these, proanthocyanidins (type A epicatechin polymers), cinnamaldehyde and cinnamic acid have been suggested to be the main active compounds responsible for the antidiabetic properties of cinnamon [3]. There were clear differences in composition between the cinnamon types (Fig. 1b), especially SC which showed low levels of the hepatotoxin coumarin. This agrees with previous reports showing negligible levels of coumarin in SC, and higher relative abundances in CC, IC and VC [18]. Coumarin levels were highest in VC followed by CC and IC. Ceylon cinnamon also showed high levels of eugenol and methoxy cinnamaldehyde as noted previously [8]. All four cinnamon types had high levels of cinnamaldehyde but VC and SC were higher than IC and CC. Some clear but more minor differences in proanthocyanidin composition were observed between the samples, with IC and SC showing higher contents.

### Total Polyphenols and Antioxidant Potential

**Fig. 2:**
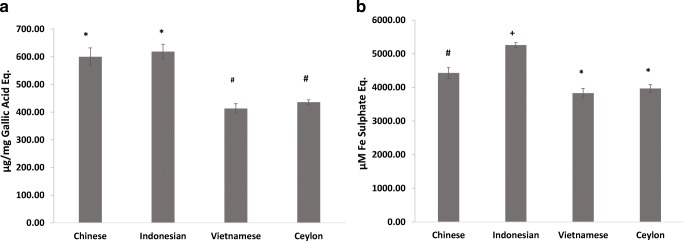
Polyphenol and antioxidant contents of cinnamon extracts. Polyphenol content (**a**) and antioxidant potential (**b**) of the cinnamon extracts. Error bars are standard deviations. Bars with different symbols are significantly different (One-way ANOVA, *P* < 0.05)

The study confirms previous evidence that cinnamon is a good source of polyphenols and antioxidants [19], although there is less species-specific comparative data in the literature. All four cinnamon types had high total polyphenol contents with statistically significant differences between types (F [3, 8]=65.7; *P* < 0.001) (Fig. 2a). CC and IC had the highest polyphenol contents (600 and 618 μg/mg GAE respectively), which were statistically similar to each other but different to VC and SC (*P* > 0.05). The polyphenol content of CC is similar to previous observations [20] but that of IC is lower than some reports [21]. The latter two cinnamons demonstrated statistically similar polyphenol contents (P > 0.05) (413 and 436 μg/mg GAE respectively) comparable to some previous reports [22] but not others [23]. Varietal differences, and growing conditions such as climate, light intensity, soil type and agronomic practices can all influence bioactive contents in plant material and explain variations in the literature [24].

Similar to previous reports [19] we observed good antioxidant properties in the cinnamon, the levels of which differed significantly between types (F [3, 8]=76.1; *P* < 0.001) (Fig. 2b). Post-hoc tests confirmed IC had significantly higher levels to the others (at 5258 μM Iron Sulphate). In comparison CC showed a significantly lower potential (4424 μM Iron Sulphate) which agrees with previous observations [21]. Vietnamese cinnamon and SC showed lower and statistically similar antioxidant potentials (3828 and 3964 μM Iron Sulphate respectively) which were also significantly different to CC and IC (*P* > 0.05).

To our knowledge this is the first instance where the polyphenol and antioxidant contents of these four cinnamon types have been compared within a single study. Whilst variations in starting material, extraction methods, analytical methods and data units preclude meaningful comparisons with the literature, the trends suggest that IC consistently has higher polyphenol and antioxidant contents, and this may be partly due to the greater proanthocyanidin content. However, CC showed low levels of proanthocyanidins and despite this showed an increased antioxidant and polyphenol level compared to the other cinnamon species, indicating the presence of other contributing compounds. Cinnamaldehydes have shown poorer effects on antioxidant potential [25] and our data appears to agree with this where VC and SC demonstrated lower FRAP values despite having substantial contents of cinnamaldehyde and methoxy cinnamaldhyde. In addition, cinnamon contains other minor compounds such as, saponins, anthraquinones, and alkaloids which could contribute to its antioxidant potential.

### *α*-Amylase and *α*-Glucosidase Enzyme Activity Inhibition

**Fig. 3:**
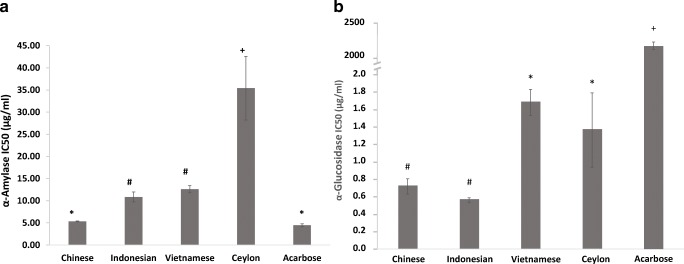
Enzyme activity inhibition by cinnamon extracts. *α*-amylase (**a**) and *α*-glucosidase (**b**) inhibition activity of the cinnamon extracts expressed as IC_50_ values. Error bars are standard deviations. Bars with different symbols are significantly different (One-way ANOVA, *P* < 0.05)

Inhibiting digestion enzyme activity is a favoured therapeutic approach for reducing postprandial glycaemia through retarding starch digestion. Compared to other spices, cinnamon is recorded to have inhibitory effects [3], however none have compared this in the four major types used commercially. This study confirms the *α*-amylase inhibitory effects of cinnamon, and showed that efficacy was significantly influenced by type ((F [4, 10]=173.5; *P* < 0.001) (Fig. 3a). Chinese cinnamon demonstrated the lowest IC_50_ (5.3 μg/mL), which was statistically similar to the clinical agent acarbose (4.2 μg/mL) suggesting it could be a natural alternative. By comparison, IC and VC showed higher IC_50_ values (10.8 and 12.6 μg/mL respectively) that were statistically similar but different to CC and SC. Ceylon cinnamon showed the highest IC_50_ at 35.4 μg/mL and was significantly different to the other three cinnamon types. This value is comparable to previous reports of IC_50_ values of up to 50 μg/mL [26] and indicates SC as having the weakest effects of the four types. We found only one study in the literature that compared enzyme inhibition rates in cinnamon types [23], and this reports higher IC_50_ values in the range of 1200- > 4000 μg/mL which may be due to the extract type used (hydro-ethanolic extracts *vs*. aqueous extracts). Studies on cinnamon show that hydro-alcoholic extracts demonstrate better functional effects than aqueous fractions [16] and this supports the trends we observed. Values for IC_50_ would also depend on the experimental conditions such as the substrate type, enzyme type, substrate/enzyme concentrations, buffer conditions, incubation temperature and extract types used, and this precludes absolute value comparisons. However, when comparing trends Adisakwattana *et al*. [23] found the lowest IC_50_ for SC followed by CC and VC, although no statistical significance was reported. The differences suggest that alcohol-soluble compounds in cinnamon such as eugenol and cinnamaldehyde may have a significant role to play in α-amylase enzyme inhibition.

Compared to the effects on *α*-amylase activity, the cinnamon extracts showed greater efficiency in inhibiting α-glucosidase activity (Fig. 3b), with a significant effect of cinnamon type (F [4, 10]=1276.6; *P* < 0.001). All four cinnamon extracts demonstrated IC_50_ values below 2 μg/mL suggesting potent inhibitory effects, particularly in comparison with acarbose which showed a value over 1000 fold greater (2175.8 μg/mL). The IC_50_ value we observed for acarbose is comparable to earlier reports [27]. Chinese cinnamon and IC showed the lowest IC_50_ values, which were statistically similar (0.7 and 0.6 μg/mL, respectively), followed by VC and SC which showed significantly higher values to the former two but statistically similar to each other (1.7 and 1.4 μg/mL, respectively). The IC_50_ for SC is within the range observed by Salehi *et al*. [26] when they examined extracts made using a variety of solvents (0.5–10 μg/mL). They observed the lowest IC_50_ in methanol extracts which suggests alcohol solvents are particularly good for cinnamon and for extracting the more potent bioactives, and this agrees with other data on cinnamon [16].

Previous studies have observed correlations between the polyphenol content of natural products and enzyme inhibitory effects [28], and similar relationships were seen in this study where polyphenol content of the extracts significantly correlated with *α* amylase (*r* = −0.59; *P* = 0.045) and *α*-glucosidase inhibition rates (*r* = −0.90; *P* < 0.001).

Overall, the data shows that Chinese cinnamon is particularly effective in inhibiting *α*-amylase while all four types are excellent at inhibiting *α*- glucosidase. A limitation to the use of Cassia type cinnamons is their high coumarin content (5800 mg/kg for CC and up to 9000 mg/kg for IC) [29], and current European guidelines recommend an intake cap of 0.1 mg/kg of body weight to avoid possible hepatotoxic and carcinogenic effects. This limits daily intakes of cassia cinnamon types to around 1 g of powder which may be insufficient to exert beneficial effects on enzyme activity. Using de-coumarinated extracts is a potential alternative.

The more pronounced effects of cinnamon on *α*-glucosidase suggests it could play a greater role in inhibiting the breakdown of sugars at the brush border, including intermediates produced from *α*-amylase activity. Since glucosidases play a central role in the cleavage of 1–6 glycosidic bonds their inhibition would reduce the breakdown of amylopectins, a starch fraction particularly associated with higher glycaemic responses. Similar to acarbose, Shihabudeen *et al* [30] found that the effects of Ceylon cinnamon on *α*-glucosidase inhibition was reversible, which is beneficial as it would only curtail digestion rate and not inhibit it. Similar work is required with other cinnamon types to determine the reversibility of their associations with digestive enzymes. Natural alternatives to acarbose could be beneficial for patients intolerant to this clinical inhibitor, however care must be taken to avoid side effects due to carbohydrate maldigestion.

### Effect of Cinnamon Extracts on Glycaemic Potential of White Bread

**Fig. 4:**
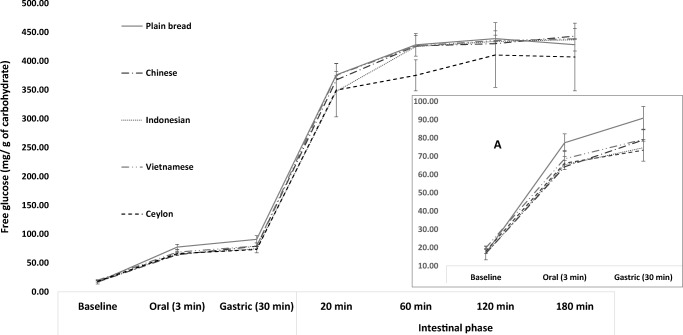
Effects of cinnamon on the glycaemic potential of white bread. Glycaemic potential of white bread measured as free glucose generated during *in vitro* gastro-intestinal digestion. Error bars are standard deviations

Cinnamon significantly affected white bread starch digestion during the oral and gastric phases of digestion (F [4, 10]=6.9; *P* = 0.006 and F [4, 10]=4.2; *P* = 0.029, respectively) but no effects were seen in the subsequent stages (Fig. 4). *Post hoc* comparisons showed that in the oral phase, CC, IC and SC statistically significantly reduced starch digestion (64.3 ± 2, 65.4 ± 1, 66.2 ± 2 mg/g of carbohydrate, respectively) compared to the plain bread control (77.4 ± 5 mg/g of carbohydrate). To our knowledge this is the first study to compare the glycaemic potential of extracts of different cinnamon types using a validated *in vitro* gastro-intestinal model. This confirms the inhibitory effects of cinnamon on *α*-amylase activity and agrees with our inhibition assay results. However, the data also shows that all three extract types curtailed starch digestion to similar degrees when used at the same concentration despite having differing amylase IC_50_ values, and this may indicate a lower importance of absolute IC_50_ values. Interestingly, SC showed a good level of inhibition despite having a relatively high IC_50_ value, and further confirms the positive antiglycaemic properties seen for this cinnamon type in previous *in vitro* and *in vivo* work [3]. Conversely, VC failed to statistically significantly curtail starch digestion (68.6 ± 5 mg/g of carbohydrate) despite an IC_50_ similar to IC. Our data agrees with reports showing poor effects of VC on *α*-amylase activity [23], however further work is required.

Compared to the plain bread control (90.9 ± 6 mg/g of carbohydrate) the presence of IC and SC significantly reduced free glucose content in the gastric phase (74.7 ± 5 and 73.3 ± 6 mg/g of carbohydrates, respectively) but not CC and VC (79.0 ± 6 and 79.3 ± 6 mg/g of carbohydrates, respectively) (*P* < 0.05, Fig. 4). Although enzyme activity should be reduced by the low pH, the buffering effects of foods can retain activity in gastric conditions for up to 30 min [31] and may explain the sustained effects of cinnamon on starch digestion. No studies have assessed the effects of gastric pH on cinnamon constituent functionality and this is an important area worthy of study.

Cinnamon did not show noteworthy effects in curtailing starch digestion during the intestinal phase. This lack of effect was not reflective of our observations in the enzyme inhibition assays and one explanation may be a reduced efficacy due to dilution effects. The volume in the intestinal digestions was 72 mL compared to 20 and 38 mL in the oral and gastric phases, respectively, and the resulting reduction in extract concentrations (to 6%) may have precluded significant inhibitory effects. Although this needs to be confirmed, its practical implications are worth considering as significant dilution of food occurs in the digestive lumen due to the secretion of fluids, and this may be influencing the functionality of natural products. Furthermore, the chyme is a complex mixture where factors such as viscosity, particle size, food and secretion compositions can affect the activity of functional compounds.

Polyphenols could influence the ratios of starch fractions such as RDS, SDS and RS [32], however this study did not show any effects of cinnamon on them (*P* > 0.05) (Table 2 in supplementary material). The RDS by definition is the glucose released in the first 20 min of intestinal digestion and is indicative of the early glycaemic response phase, SDS is that digested between 20 and 120 min and RS that remains undigested at the end of 120 min. Therefore, a starch profile favouring higher SDS and RS contents could be indicative of a lower glycaemic response. Comparative data is unavailable as the effects of cinnamon on starch fractions has not been previously measured, and therefore further confirmatory studies are required.

**Fig. 5:**
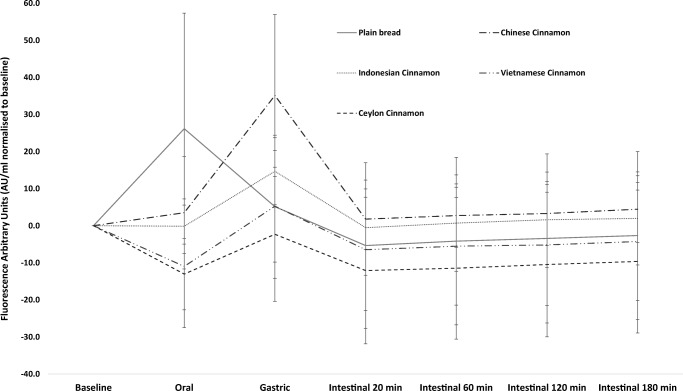
Generation of advanced glycation end-products during gastro-intestinal digestion. Values are corrected for baseline. Error bars are standard deviations

We also measured AGEs in digesta samples as a qualitative measure to assess effects of cinnamon on the generation of Maillard reaction products during gastro-intestinal digestion. The analytical method used in the current study only quantified fluorescent AGEs which are a less stable group of reaction products. This is evident from the large standard deviations observed in the data (Fig. 5), and therefore is a method best used for obtaining indicative data. The results suggest that all four cinnamon types retard AGEs formation in the oral phase of digestion compared to the control. Indonesian cinnamon, VC and SC showed the best effects in reducing AGEs, and the latter in particular. This agrees with previous data showing a beneficial effect of SC on AGEs [33]. Interestingly all cinnamons showed a peak in AGEs in the gastric phase contrary to what could be expected. The effect of pH on AGEs generation is complex and although the rate of the initial step of the Maillard reaction with the amino groups is decreased there is an increased reactivity with carbonyl groups of reducing carbohydrates at low pH [34]. The beneficial effects of cinnamon on AGEs have been ascribed to catechin, epicatechin and particularly proanthocyanidins acting as reactive carbonyl scavengers [35]. Loss of the inhibitory activity of the extracts in the gastric phase may be due to changes in molecular conformation at the lower pH affecting the susceptibility for glycation and also the types of products formed [36].

The cinnamon concentrations tested in the *in vitro* digestions were at practically relevant levels corresponding to 0.07, 0.2, 0.4 and 0.6 g of CC, IC, VC and SC bark powders, respectively. These amounts are approximately equivalent to consuming 640 mg of cinnamon extract with an 80 g portion of white bread. The promising effects observed with this relatively low level of supplementation suggests room for safely increasing levels and possibly enhancing physiological effects, however this remains to be confirmed in dose response studies.

To our knowledge, this is the first study to systematically compare the four most commercially relevant cinnamon types for some of their anti-hyperglycaemic properties. The distinct phytochemical profiles observed for the four types highlight the importance of considering cinnamon on a species-specic basis rather than as a generic whole. All four cinnamon types showed strong evidence of digestive enzyme inhibition and confirms this as a mechanistic pathway for potential antidiabetic effects. Chinese cinnamon showed the strongest activity against *α*-amylase, and all four types potently inhibited *α*-glucosidase activity. At practically relevant concentrations cinnamon extracts, particularly Indonesian and Ceylon types retarded starch digestion in the oral and gastric phases of gastro-intestinal digestion, however none showed effects during small intestinal digestion. Synthesising all the findings of the study Ceylon cinnamon showed the best overall potential in terms of bioactive and anti-nutrient profiles, enzyme inhibitory activity and effects on starch digestion.

## Electronic supplementary material

ESM 1 (DOCX 17 kb)
