# Supplementary material for: Cinnamon Shows Antidiabetic Properties that Are Species-Specific: Effects on Enzyme Activity Inhibition and Starch Digestion
Source: Plant Foods Hum Nutr. 2019 Aug 1;74(4):544–52. doi: 10.1007/s11130-019-00760-8 (PMC6900266; doi:10.1007/s11130-019-00760-8)
Supplement: Supplementary file 1 — (DOCX 17 kb) [file 11130_2019_760_MOESM1_ESM.docx]

Supplementary material

**Table 1:** Composition of digestion fluids

|  | **Saliva** | **Gastric fluid** | **Intestinal fluid** |
| --- | --- | --- | --- |
| Inorganics |  |  |  |
| KCl | 15.1 mM | 6.9 mM | 6.8 mM |
| KH_2_PO4 | 3.7 mM | 0.9 mM | 0.8 mM |
| NaHCO_3_ | 13.6 mM | 25 mM | 85 mM |
| NaCl | - | 47.2 mM | 38.4 mM |
| MgCl_2_.6H_2_O | 0.15 mM | 0.12 mM | 0.33 mM |
| (NH_4_)2CO_3_ | 0.06 mM | 0.5mM | - |
| CaCl_2_.2H_2_O | 1.5 mM | 0.15 mM | 0.6 mM |
| HCl | - | 8 mM | - |
| NaOH | - | - | 2 mM |
| Enzymes |  |  |  |
| α- amylase | 0.5 mg/ml | - | - |
| Pepsin | - | 7 mg/ml | - |
| α- glucosidase  (260 U/ml) | - | - | 2 U/ml |
| Pancreatin | - | - | 1.5 mg/ml |
| Bile | - | - | 3.75 mg/ml |
| pH | 7 | 2 | 7 |

Inorganic salt solutions were prepared as stock solutions, and final digestive fluids were made fresh on the day with the addition of reconstituted enzymes. Formualtions based on [10]

**Table 2:** RDS, SDS and RS contents after gastro-intestinal digestion of white bread

|  | RDS | SDS | RS |
| --- | --- | --- | --- |
| Control | 338.5±5 | 56.7±9 | 324.08±5 |
| Chinese cinnamon | 330.6±5 | 56.7±9 | 332.7±9 |
| Indonesian cinnamon | 312.8±21 | 78.0±10 | 329.2±14 |
| Vietnamese cinnamon | 337.9±18 | 54.4±4 | 327.7±15 |
| Ceylon cinnamon | 314.8±42 | 54.8±13 | 350.4±50 |

RDS=Rapidly Digestible Starch, SDS=Slowly Digestible Starch, RS=Resistant Starch; Values are means ± SD (n=4)
